# Analysis of Food Safety Knowledge and Practices Among Food Handlers in Restaurants and Street Food Markets in Dhaka, Bangladesh: A Cross-Sectional Study

**DOI:** 10.1155/ijfo/5369920

**Published:** 2025-01-07

**Authors:** Md. Shamsur Rahman, Md. Shameem Hossain, Md. Fuad Hasan Pulok

**Affiliations:** Department of Nutrition and Food Engineering, Faculty of Health and Life Sciences, Daffodil International University, Dhaka 1216, Bangladesh

**Keywords:** cross-contamination, foodborne illnesses, food safety knowledge, restaurant food handlers

## Abstract

This study is aimed at analyzing food safety knowledge and practices among food handlers in restaurants and street food markets in Dhaka, Bangladesh. Inadequate food handling practices remain a major worldwide health problem and are one of the main causes of food-related diseases. In Bangladesh, where the restaurant business is expanding quickly, food safety must be upheld to stop foodborne illness outbreaks. A cross-sectional study involving 300 street food vendors and restaurant workers was conducted through in-person interviews using a structured questionnaire. Descriptive and inferential statistical methods were employed to analyze the data. The study revealed that while most workers understood basic hygiene practices, critical knowledge gaps persisted. Male workers demonstrated lower food safety knowledge than females (*p* ≤ 0.01), and higher knowledge scores were associated with both greater education (*p* ≤ 0.001) and experience (*p* ≤ 0.05). Workers in fast food establishments showed higher knowledge levels (*p* ≤ 0.001), and knowledge was more strongly linked to job responsibilities than to training (*p* ≤ 0.001). In terms of practices, women adhered more closely to food safety guidelines than men (*p* ≤ 0.05). Age, education, and experience had no significant effects on practice adherence, though fast-food workers exhibited higher compliance (*p* ≤ 0.05). Training and job responsibilities showed no significant effects on food safety practices. The results demonstrate that there are disparities in food handlers' understanding of food safety, with gaps primarily in the areas of cross-contamination avoidance, personal hygiene, and temperature control. Furthermore, observed practices suggest suboptimal adherence to advised food safety protocols.

## 1. Introduction

Bangladesh's restaurant industry has seen remarkable growth in recent years (2024), offering a diverse array of dining options that cater to various tastes and budgets. This includes traditional Bangladeshi restaurants serving authentic cuisine, fast food chains providing quick and affordable meals, fine dining establishments with gourmet menus and premium service, and street food vendors offering a wide variety of snacks and meals at economical prices. Given this expansion, it is crucial to prioritize food safety to prevent foodborne illnesses. To ensure food safety in restaurants, food handlers are crucial [1]. The knowledge and practices of food handlers in Bangladeshi restaurants in relation to food safety have only been extensively examined in a limited number of studies. Understanding the current situation is crucial to developing targeted solutions to improve food safety [2]. Global public health is seriously threatened by foodborne infections, of which there are an estimated 600 million cases reported yearly and 420,000 fatalities worldwide [3]. Since incorrect food handling practices are the primary source of foodborne illnesses, it is imperative to uphold food safety across the whole food supply chain, from production to consumption [4]. Food handlers, particularly those employed by restaurants and other food service businesses, are vital in this aspect since they directly affect the safety of the food that is served to patrons [5].

Bangladesh, a densely populated country in South Asia, is experiencing rapid urbanization and economic growth, leading to an expansion of its restaurant industry [6]. To safeguard the public's health as more and more people, dine out, restaurants are required to adhere to stringent food safety laws. However, foodborne illness outbreaks are often reported in Bangladesh, which raises questions over the prevalence of these disorders [7]. It is crucial to comprehend the degree of food safety knowledge and practices held by food handlers in restaurants in Bangladesh in order to spot any gaps and put specific interventions in place to reduce the risk of foodborne infections. In Bangladesh, the most common foodborne diseases are hepatitis and enteric fever, followed by diarrheal disorders. According to research conducted by the Institute of Epidemiology, Disease Control, and Research (IEDCR) in Dhaka, acute watery diarrhea is the most prevalent outcome of food poisoning, with over 0.28 million cases reported in 2015. Annually, foodborne hepatitis affects approximately 500 people, while enteric fever impacts around 30,000 individuals [8].

Few studies have specifically examined food handlers in restaurants in Bangladesh, despite a range of global research on food safety procedures. While several studies in Bangladesh have evaluated food safety knowledge and practices among food handlers from various professional backgrounds, findings indicate a general lack of adequate knowledge and practices, particularly among those in more informal settings. One investigation highlighted inadequate food safety knowledge and poor hygiene practices among street food vendors in Dhaka City [9]. Similarly, findings from research on Bangladeshi meat handlers indicated poor food safety knowledge and practices [8]. An evaluation of chicken vendors in Dhaka's wet markets revealed insufficient knowledge and poor practices related to food safety and pathogens [10]. Another assessment of fish farmers and food handlers in Noakhali showed that their understanding of food safety and hygiene was inadequate [11]. Additionally, an analysis of school-based street food vendors in Dhaka found that over two-thirds lacked sufficient knowledge of food safety issues relevant to children [12].

In contrast, food handlers in more formal settings, such as hospitals and food industries, generally demonstrated better food safety knowledge and practices. Research on hospital food service staff indicated moderate knowledge levels but high positive attitudes and practices regarding food safety [8]. Observations in the baking industry showed that trained workers scored significantly higher in food safety knowledge, attitudes, and practices compared to their newly appointed, untrained counterparts [13]. Similarly, food handlers in the biscuit industry displayed excellent knowledge, attitudes, and practices related to food hygiene and sanitation [14].

This cross-sectional study is aimed at filling a critical gap by evaluating food safety procedures and practices among restaurant food handlers in Bangladesh. Beyond public health implications, it seeks to provide evidence-based recommendations to improve food safety standards, boosting consumer confidence and supporting economic growth [15]. High food safety standards are crucial not only for public health but also for the reputation and sustainability of the restaurant industry [16]. As consumers increasingly value safe food, businesses prioritizing safety are better positioned to earn customer trust and drive industry competitiveness [17].

The study assesses compliance with food safety protocols in food preparation, storage, and service among Bangladeshi restaurant food handlers, identifying knowledge gaps in areas such as temperature control, personal hygiene, and cross-contamination prevention [12]. It also examines the link between food handlers' knowledge and practices and demographic factors like age, gender, education, and experience. The ultimate goal is to reduce foodborne illnesses and protect public health in Bangladesh [18], while providing evidence-based recommendations for policy improvements in the restaurant sector.

## 2. Methodology

### 2.1. Study Design and Sampling Procedure

**Figure 1:**
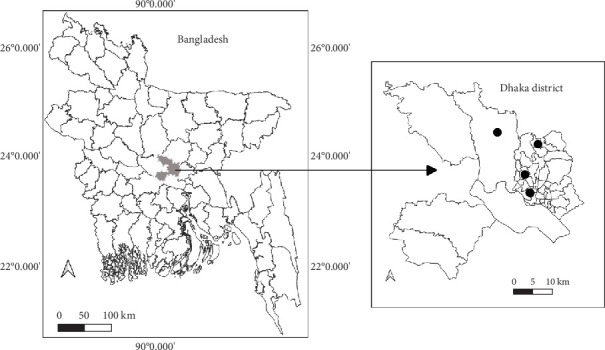
Study locations in Dhaka, Bangladesh (marked by circles).

This cross-sectional study was conducted from January to May 2024 in four urban areas of the Dhaka district—Dhanmondi, Mirpur, Uttara, and Savar (Figure 1)—utilizing a convenience sampling technique. To determine the sample size, the conventional formula for cross-sectional studies was applied:

 $$\begin{array}{c} n=\frac{z^{2}p\left(1-p\right)}{d^{2}} \end{array}$$

where *n* is the required sample size, *z* is the *z*-score corresponding to the desired confidence level, *p* is the estimated proportion of the population, and *d* is the margin of error. Based on a study where 76% of food handlers were reported to have poor food safety knowledge and with a confidence level of 95% (*z* = 1.96) and a margin of error of ± 5% (*d* = 0.05), the required sample size was calculated to be 281 ([19]). Considering potential missing data or incomplete surveys, the final sample size for this study was adjusted to 300. The survey included food handlers from different food service establishments including casual dining, restaurant, fast-food outlets, and street food vendors.

### 2.2. Data Collection

A structured questionnaire was developed to gather data through in-person interviews based on prior research. It was subsequently revised based on feedback from experts in the field for internal validation. At the time of data collection, the Bengali translation of the English version of the questionnaire was used. A multilingual translator completed the first translation from English to Bengali, and another bilingual member of the study crew verified it twice. An impartial bilingual research staff member performed back-translation in order to guarantee consistency and remove bias. Finally, a pretest of the questionnaire was conducted with 10 randomly chosen food handlers (*n* = 10) in order to verify its appropriateness, lucidity, and interview duration requirements. After explaining the goal of the research to each participant, data collectors obtained verbal or written consent from those who want to take part. Verbal consent was preferred for illiterate participants and was documented by logging each participant's name, date, and consent type. Each interview took approximately 20–25 min. Participants' responses were anonymized through coding of the questionnaires.

### 2.3. Study Variables and Measures

A modified version of a questionnaire from earlier research [20, 21] was used. Three separate sections made up the questionnaire: Part 1: contained information on demographics such as age, sex, education level, years of food handling experience, kind of restaurant, and area of responsibility. Section 2: Knowledge on food safety was examined (10 questions) [22]. Part 2: The food handlers were asked about their knowledge regarding preparation and food conservation, foodborne diseases, their personal hygiene, cross-contamination, and time and temperature control of foodstuffs, and each question consisted of three optional answers: true, false, and I do not know (Table 1). Each correct response earned one point, while incorrect or “I do not know” responses scored zero points. The total food safety knowledge score ranged from 0 to 10. Scores of 5 or above were classified as a good level of knowledge, while scores below 5 were classified as a poor level of knowledge. Part 3: taking food safety into consideration. Eleven questions about food handling practice were given, with five possible responses for each: never, seldom, occasionally, often, and always. These inquiries centered on concerns about microbes, foodborne illnesses, cross-contamination, personal hygiene, and hygiene procedures. A five-point ordinal scale was used to assess the level of practices: *never* (0), *rarely* (1), *sometimes* (2), *frequently* (3), and *always* (4). Items 1, 2, 8, and 11 were the four questions for which reverse scoring was used. The overall practice scores were in the range of 0–44, with a score of less than 22 indicating poor practice and a score of 22 or more indicating good practice.

### 2.4. Statistical Analysis

For data analysis, SPSS software Version 20.0 for Windows was used. Descriptive statistics were applied to summarize the demographic characteristics, knowledge levels, and practices of food handlers. The chi-square test was used to measure the association of different factors with food safety knowledge and practice, and a *p*≤ 0.05 was considered statistically significant for all analyses.

## 3. Result and Discussions

### 3.1. Result

#### 3.1.1. Demographic Characteristics

The sociodemographic data (Table 2) of the participants (*N* = 300) showed that the majority were male (86.3%). Most participants were between 35 and 44 years old (34.3%), followed closely by those aged 25–34 (29.7%). Educational attainment was primarily primary (36.7%) or secondary (27.3%), though 19% were illiterate. Most workers were employed in casual dining (45.9%) and fast food restaurants (21.7%), with food servers (30.7%) and cooks (27.3%) being the most common roles. Of the participants, 36.0% had 3–5 years of experience as food handlers, though only 1.7% had food safety training.

#### 3.1.2. Food Safety Knowledge

Table 1 summarizes the responses of food handlers to questions assessing their food safety knowledge. While most participants understood key practices such as the importance of washing hands (58.7%) and the need for food handlers with infectious diseases to avoid working (74.7%), there were notable deficiencies in other critical areas. A large proportion were unaware of the correct storage practices for raw and ready-to-eat foods, with 62% unsure about the risks of storing them together and only 12% identifying this as incorrect. Understanding of temperature control was particularly weak, with 89% unaware of the danger zone for food temperatures. Although 73.3% recognized the need to refrigerate leftovers promptly, there was confusion about safe equipment use, as 60% did not know whether the same cutting board could be used for vegetables and raw meat. Only 21.3% correctly understood cross-contamination risks associated with cutting boards, and only 6.7% correctly identified the temperature danger zone.

#### 3.1.3. Food Safety Practice

Table 3 shows the responses from food handlers regarding their food safety practices. The data reveals that a significant number of food handlers engage in potentially unsafe practices, such as consuming food or beverages inside food processing areas (40.7% never do this, but 36.3% do it rarely) and smoking inside these areas (68.7% never do this). Most handlers wash their hands before and after using gloves (73.3% always) and after handling waste (79%), though handwashing after smoking, sneezing, or coughing is less consistent (87% always). All participants reported using sanitation after using the toilet. However, a substantial number of handlers do not consistently use hairnets or caps (41.3% never and 17.7% rarely), and many use rings, watches, or necklaces while processing food (46.3% sometimes). Compliance with dress code is generally high (59.7% always), and most handlers clean equipment properly (87.7% always). Handling money while processing food is common, with 49% of respondents doing this occasionally.

#### 3.1.4. Factors Associated With Food Safety Knowledge

Table 4 examines factors associated with food safety knowledge among 300 food handlers using a chi-square test. Gender shows a significant difference (*p* ≤ 0.01), with more males (63.7%) having poor knowledge compared to females (36.6%). Education level strongly affects knowledge (*p* ≤ 0.001), with illiterate handlers and those with only primary school education showing poorer knowledge, while all handlers with a bachelor's degree demonstrated good knowledge. Experience also influences knowledge (*p* ≤ 0.05), with handlers having over 5 years of experience showing better knowledge (51.2% good). The type of workplace is significant (*p* ≤ 0.001), with fast-food restaurant handlers showing better knowledge compared to those in street food vending and other settings. Job responsibility also affects knowledge (*p* ≤ 0.001), with chefs showing the highest percentage of good knowledge (74.3%), while food servers had the lowest (30.4%). Food safety training shows no significant difference (*p* > 0.05), indicating similar knowledge levels regardless of training.

#### 3.1.5. Factors Associated With the Food Safety Practice

Table 5 presents the association between the food safety practices of food handlers and various factors, based on a chi-square test analysis. Gender shows a significant association with food safety practices (*p* ≤ 0.05), with a higher proportion of females (26.8%) demonstrating good food safety practices compared to males (13.1%). The type of workplace also significantly influences food safety practices (*p* ≤ 0.05), with those working in casual dining settings showing better practices (19.6%) compared to street food vendors, who exhibited the lowest level of good practices (2.1%). Other factors, including age, education level, experience, job responsibility, and food safety training, did not show a significant association with food safety practices (*p* > 0.05).

### 3.2. Discussion

The findings of this study reveal significant gaps in food safety knowledge and practices among food handlers, highlighting the need for targeted interventions to enhance standards in the food industry. Demographic data indicate a predominantly male workforce with limited education and minimal formal food safety training. Despite many food handlers working in structured environments like casual dining and fast-food restaurants, a considerable number still engage in unsafe food handling practices, underscoring the need for improved education and training initiatives. Although basic food safety practices, such as handwashing and avoiding work when ill, are widely recognized, substantial deficiencies remain in critical areas like temperature control, cross-contamination prevention, and proper food storage. These findings align with other studies that report low awareness of temperature danger zones and improper food storage among food handlers, with one study noting only 15% of handlers correctly identifying the temperature danger zone, similar to the 6.7% observed in this study [23, 24]. Misconceptions about cross-contamination and inadequate food safety knowledge continue to be common issues that contribute to poor food handling practices [25].

The data also show that food handlers frequently engage in unsafe behaviors, such as consuming food, beverages, or smoking in food processing areas. Similar patterns of poor hygiene practices have been reported, including inconsistent use of personal protective equipment like gloves and hairnets. In some studies, around 42% of food handlers failed to consistently use hairnets, comparable to the 41.3% observed in this study [26]. Additionally, wearing personal accessories such as rings and watches was prevalent among food handlers, posing contamination risks and reflecting the need for stricter enforcement of hygiene protocols and continuous education on proper food safety behavior [27].

Gender and workplace type significantly influence food safety knowledge and practices. Evidence suggests that female food handlers often demonstrate better compliance with food safety protocols than their male counterparts, potentially due to differences in adherence or cultural norms, consistent with the observations in this study [28]. The type of workplace also impacts practices, with those in structured environments like casual dining showing better compliance compared to handlers in less regulated settings like street vending [29]. However, contrasting findings suggest that experience and formal training significantly improve food safety practices, whereas this study found that training did not significantly affect knowledge or practices, with only 1.7% of participants having received training. This discrepancy underscores the need for more effective training interventions in Bangladesh, focusing on quality and content improvements [30].

Overall, the study indicates that while factors like gender, workplace, and job responsibility influence food safety knowledge and practices, pervasive gaps across demographic groups emphasize the urgent need for comprehensive, targeted training. Efforts should prioritize enhancing practical food safety education, particularly for street vendors and those with lower educational backgrounds, and enforce stricter oversight of hygiene practices to reduce food handling risks.

### 3.3. Limitation of the Study

The cross-sectional design of the study limits the ability to establish causal relationships. Convenience sampling may have introduced selection bias, and self-reported data may be subject to recall or social desirability bias. The study's focus on four locations in Dhaka district limits generalizability, and the lack of observational data may have affected the accuracy of self-reported practices. Additionally, a larger sample size could have provided greater statistical power. These limitations should be considered when interpreting the findings and planning future research.

## 4. Conclusion

The study reveals significant gaps in food safety knowledge and practices among Bangladeshi food handlers, exacerbated by limited education, minimal training, and unsafe practices like consuming food or beverages in food processing areas. While gender and workplace type influence food safety, factors such as age, education, experience, and job responsibility do not significantly affect practices. To improve food safety, Bangladesh needs targeted, practical training programs, enhanced training quality, stricter oversight of hygiene protocols, and increased cultural awareness. Implementing these recommendations can significantly enhance food safety standards and protect public health in Bangladesh.

## Figures and Tables

**Table 1:** Summary of questions and responses for assessment of food safety knowledge of food handlers (*N* = 300).

**Statements**	**Responses, ** **n** ** (%)**		
	**True**	**False**	**Do not know**
Washing hands with soap and water before work reduces the risk of food contamination.	176 (58.7)	5 (1.7)	119 (39.7)
Wearing hairnets, gloves, and masks is not important for food safety.	54 (18)	214 (71.3)	32 (10.7)
Eating and drinking in the workplace is not a risk for food contamination.	48 (16)	73 (24.3)	179 (59.7)
Food should be cooked to a minimum internal temperature of 74°C to kill harmful bacteria.	174 (58)	8 (2.7)	118 (39.3)
A food handler with an infectious disease should take leave and should not work on food preparation activities.	224 (74.7)	6 (2)	70 (23.3)
Raw meat can be stored with ready-to-eat foods in the refrigerator together.	78 (26)	36 (12)	186 (62)
Leftovers should be refrigerated within 2 h of being cooked.	220 (73.3)	14 (4.7)	66 (22)
It's safe to use the same cutting board for vegetables and raw meat as long as you wash it between uses.	64 (21.3)	56 (18.7)	180 (60)
Jewelry should not be worn by food handlers when preparing food, as they can harbor dirt and bacteria.	170 (56.7)	15 (5)	115 (38.3)
The danger zone for food temperatures is between 40°F (4.4°C) and 140°F (60°C).	20 (6.7)	13 (4.3)	267 (89)

**Table 2:** Sociodemographic characteristics of study participants (*N* = 300).

**Variable**	**Categories**	**Frequency**	**Percentage**
Gender	Male	259	86.30
	Female	41	13.70

Age (years)	< 18	10	3.30
	18–24	66	22.0
	25–34	89	29.7
	35–44	103	34.3
	> 44	32	10.7

Education level	Illiterate	57	19.0
	Primary school	110	36.7
	High school graduate	82	27.3
	Some college/technical training	27	9.0
	Bachelor's degree and higher	24	8.0

Year of experience as a food handler	< 1	23	7.7
	1–2	87	29.0
	3–5	108	36.0
	> 5	82	27.3

Type of working place	Fast food restaurant	65	21.7
	Casual dining	138	45.9
	Fine dining	38	12.7
	Street food vendors	48	16.0
	Others	11	3.7

Type of responsibility	Chef	35	11.7
	Helper	55	18.3
	Food server	92	30.7
	Cook	82	27.3
	Others	36	12.0

Food safety training	Yes	5	1.7
	No	295	98.3

**Table 3:** Summary of questions and responses for assessment of food safety practice of food handlers (*N* = 300).

**Questions**	**Responses, ** **n** ** (%)**				
	**Never**	**Rarely**	**Sometime**	**Often**	**Always**
Do you consume food or beverages inside food processing areas?	122 (40.7)	108 (36.0)	61 (20.3)	2 (0.7)	7 (2.3)
Do you smoke inside food processing areas?	206 (68.7)	52 (17.3)	38 (12.7)	1 (0.3)	3 (1.0)
Do you wash your hands before and after the use of gloves?	1 (0.3)	6 (2)	25 (8.3)	48 (16)	220 (73.3)
Do you wash your hands after handling waste or garbage?	1 (0.3)	3 (1)	11 (3.7)	48 (16)	237 (79.0)
Do you use sanitation to wash hands after using toilet?	0 (0.0)	0 (0)	0 (0.0)	0 (0)	300 (100.0)
Do you wash your hands after smoking, sneezing, or coughing?	1 (0.3)	1 (0.3)	4 (1.3)	33 (11)	261 (87.0)
Do you use a hairnet or a cap while working for food safety?	124 (41.3)	53 (17.7)	82 (27.3)	12 (4)	29 (9.7)
Do you use rings, watches, or necklaces while you process the food?	112 (937.3)	35 (11.7)	139 (46.3)	7 (2.3)	7 (2.3)
Do you follow the dress code when you process the food?	179 (59.7)	74 (24.7)	36 (12.0)	2 (0.7)	9 (3.0)
Do you properly clean all equipment?	2 (0.7)	3 (1)	10 (3.3)	22 (7.3)	263 (87.7)
Do you handle money while processing food?	147 (49)	51 (17)	52 (17.3)	20 (6.7)	30 (10.0)

**Table 4:** Factors associated with the food safety knowledge of food handlers (*N* = 300).

**Variables**	**Food safety knowledge level**		**p** ** value**
	**Good**	**Poor**	
Gender			
Male	94 (36.3)	165 (63.7)	0.002^**^
Female	26 (63.40)	15 (63.6)	
Age (years)			
< 18	3 (30.0)	7 (70.0)	0.169
18–24	21 (31.8)	45 (68.2)	
25–34	39 (43.8)	50 (56.2)	
35–44	39 (37.9)	64 (62.1)	
> 44	18 (56.3)	14 (43.8)	
Education level			
Illiterate	15 (26.3)	42 (73.7)	≤ 0.001^***^
Primary school	35 (31.8)	75 (68.2)	
High school graduate	38 (46.3)	44 (53.7)	
College/technical training	8 (29.6)	19 (70.4)	
Bachelor's degree and higher	24 (100)	0 (0.0)	
Experiences			
< 1 year	5 (21.7)	18 (78.3)	0.037^*^
1–2 years	30 (34.5)	57 (65.5)	
3–5 years	43 (39.8)	65 (60.2)	
> 5 years	42 (51.2)	40 (48.8)	
Type of working place			
Fast food restaurant	36 (55.4)	29 (44.6)	≤ 0.001^***^
Casual dining	61 (44.2)	77 (55.8)	
Fine dining	15 (39.5)	23 (60.5)	
Street food vendors	7 (14.6)	41 (85.4)	
Others	1 (9.1)	10 (90.9)	
Responsibility			
Chef	26 (74.3)	9 (25.7)	≤ 0.001^***^
Helper	21 (38.2)	34 (61.8)	
Food server	28 (30.4)	64 (69.6)	
Cook	32 (39.0)	50 (61.0)	
Others	13 (36.1)	23 (63.9)	
Food safety training			
Yes	2 (40.0)	3 (60.0)	1.000
No	118 (40.0)	177 (60.0)	

^*^Statistical significance at *p* ≤ 0.05.

^**^Statistical significance at *p* ≤ 0.01.

^***^Statistical significance at *p* ≤ 0.001.

**Table 5:** Factors associated with the food safety practice of food handlers (*N* = 300).

**Variables**	**Food safety practice level**		**p** ** value**
	**Good**	**Poor**	
Gender			
Male	34 (13.1)	225 (86.9)	0.032^*^
Female	11 (26.8)	30 (73.2)	
Age (years)			
< 18	2 (20.0)	8 (80.0)	0.888
18–24	10 (15.2)	56 (84.8)	
25–34	14 (15.7)	75 (84.3)	
35–44	16 (15.5)	87 (84.5)	
> 44	3 (9.4)	29 (90.6)	
Education level			
Illiterate	9 (15.8)	48 (84.2)	0.178
Primary school	16 (14.5)	94 (85.5)	
High school graduate	15 (18.3)	67 (81.7)	
College/technical training	5 (18.5)	22 (81.5)	
Bachelor's degree and higher	0 (0.0)	24 (100.0)	
Experiences (years)			
< 1	4 (17.4)	19 (82.6)	0.527
1–2	9 (10.3)	78 (89.7)	
3–5	18 (16.7)	90 (83.3)	
> 5	14 (17.1)	68 (82.9)	
Type of working place			
Fast food restaurant	10 (15.4)	55 (84.6)	0.014^*^
Casual dining	27 (19.6)	111 (80.4)	
Fine dining	7 (18.4)	31 (81.6)	
Street food vendors	1 (2.1)	47 (97.9)	
Others	0 (0.0)	11 (100.0)	
Responsibility			
Chef	3 (8.6)	32 (91.4)	0.764
Helper	8 (14.5)	47 (85.5)	
Food server	17 (18.5)	75 (81.5)	
Cook	12 (14.6)	70 (85.4)	
Others	5 (13.9)	31 (86.1)	
Food safety training			
Yes	1 (20.0)	4 (80.0)	1.000
No	44 (14.9)	251 (85.1)	
Food safety knowledge			
Good	15 (12.5)	105 (87.5)	0.318
Poor	30 (16.7)	150 (83.3)	

^*^Statistical significance at *p* < 0.05.

## Data Availability

The data that support the findings of this study are available from the corresponding author upon reasonable request.
